# Targeting GLI by GANT61 involves mechanisms dependent on inhibition of both transcription and DNA licensing

**DOI:** 10.18632/oncotarget.13376

**Published:** 2016-11-15

**Authors:** Ruowen Zhang, Jiahui Wu, Sylvain Ferrandon, Katie J. Glowacki, Janet A. Houghton

**Affiliations:** ^1^ Department of Oncology, Division of Drug Discovery, Southern Research, Birmingham, AL, USA; ^2^ Lerner Research Institute, Cleveland Clinic, Cleveland, OH, USA

**Keywords:** GLI, GANT61, transcription, DNA licensing

## Abstract

The GLI genes are transcription factors and in cancers are oncogenes, aberrantly and constitutively activated. GANT61, a specific GLI inhibitor, has induced extensive cytotoxicity in human models of colon cancer. The FOXM1 promoter was determined to be a transcriptional target of GLI1. In HT29 cells, inhibition of GLI1 binding at the GLI consensus sequence by GANT61 led to inhibited binding of Pol II, the pause-release factors DSIF, NELF and p-TEFb. The formation of R-loops (RNA:DNA hybrids, ssDNA), were reduced by GANT61 at the FOXM1 promoter. Pretreatment of HT29 cells with α-amanitin reduced GANT61-induced γH2AX foci. Co-localization of GLI1 and BrdU foci, inhibited by GANT61, indicated GLI1 and DNA replication to be linked. By co-immunoprecipitation and confocal microscopy, GLI1 co-localized with the DNA licensing factors ORC4, CDT1, and MCM2. Significant co-localization of GLI1 and ORC4 was inhibited by GANT61, and enrichment of ORC4 occurred at the GLI binding site in the FOXM1 promoter. CDT1 was found to be a transcription target of GLI1. Overexpression of CDT1 in HT29 and SW480 cells reduced GANT61-induced cell death, gH2AX foci, and cleavage of caspase-3. Data demonstrate involvement of transcription and of DNA replication licensing factors by non-transcriptional and transcriptional mechanisms in the GLI-dependent mechanism of action of GANT61.

## INTRODUCTION

The GLI genes, GLI1 and GLI2, are transcription factors that regulate target genes at the distal end of the canonical Hedgehog (HH) signaling pathway (SHH- > PTCH- > SMO- > GLI). Their expression in these processes is tightly regulated, with GLI1 and GLI2 being activated and deactivated at critical times during regulation of the normal cellular processes of embryogenesis, tissue patterning, and differentiation [1-3]. Both GLI1 and GLI2 are oncogenes, can induce transformation and tumorigenesis [4-6], and are constitutively activated in many types of human cancers [1, 7]. Oncogenic pathways, including KRAS/BRAF that occur in high frequency in colon cancer [8-10], circumvent the canonical HH-GLI axis by converging on and further driving GLI to a higher activating state in tumor cells, promoting cellular proliferation, tumor progression and survival [7, 11-13]. Thus, potential targets upstream of GLI are bypassed, including SMO, and GLI becomes a nodal point of activation for oncogenic KRAS signaling.

GLI1 and GLI2 are zinc finger proteins, one of the most common DNA-binding motifs in eukaryotic transcription factors [14, 15]. They are transcriptional activators, binding at promoters to GACCACCCA-like consensus sequences [1, 16, 17]. From genetic and biochemical studies, we and others suggest that GLI2 is the primary mediator of HH signaling, which activates GLI1 to transcriptionally regulate target genes and augment HH signaling quantitatively as well as qualitatively [1, 17-19]. GANT61, an experimental agent in preclinical studies, was originally identified in a cell-based screen for small molecule inhibitors of GLI1-mediated transcription [20]. This agent has induced extensive cytotoxicity in human models of colon cancer [21-23], suggesting that GLI is a critical target in colon cancer cell survival, and in other cancers where GLI is constitutively activated and/or an oncogenic KRAS-GLI axis drives proliferation. We have previously demonstrated that GANT61 inhibits GLI-dependent transcription in these models, binding specifically to GLI proteins and not to DNA or other transcription factors [24].

Binding of transcription factors to DNA takes place during all phases of the cell cycle, except during mitosis [25, 26]. Recruitment of the RNA Pol II transcription initiation apparatus to promoters by specific DNA-binding transcription factors, including GLI, is recognized as the first key regulatory step in selective transcription at eukaryotic genes [27-29]. This forms the pre-initiation complex (PIC) [30]. Tight-binding of Pol II at the PIC allows the DNA double helix to unwind, forming a transcription bubble. One DNA strand becomes the template for complementary RNA base-pairing with ribonucleotides, joined by Pol II. Following initiation of RNA synthesis, pausing of Pol II occurs during early elongation [27, 28, 30-35]. The pause factors DRB-sensitivity inducing factor (DSIF [Spt5], [36]) and negative elongation factor (NELF, [37]) cooperate to generate a Pol II pause just downstream of the transcription start site (TSS) [29]. Pausing maintains an open and accessible promoter structure to facilitate binding of additional regulatory components of the transcription machinery [29], and requires further signals to elicit the transition to a productive elongation complex [29, 38, 39]. Release of paused Pol II requires the kinase activity of positive transcription elongation factor b (p-TEFb [cdk^9^], [34, 36]), which phosphorylates the repressive DSIF-NELF complex, causing NELF to dissociate from Pol II while DSIF-p and p-TEFb travel with Pol II during elongation [29, 39, 40]. A second site by which specific transcription factors can regulate transcription is the release of Pol II pausing mediated by p-TEFb. Two transcription factors, c-Myc [30] and NF-κB [41], both key regulators of cellular proliferation, can bind p-TEFB to stimulate the elongation of transcription; binding occurs through interaction *via* the BET protein BRD4 (reviewed in [42, 43]). Following inhibition of GLI-dependent transcription and stalling of Pol II, the dynamic of Pol II, GLI, DSIF, NELF and P-TEFb on promoter DNA is unknown.

Regions rich in CG nucleotides, CpG islands, are approximately 1kb long, are free of methylation [44], and occur in the promoter regions of human genes [45]. This GC skew occurs in the region of the TSS, ranging from -500 to +1500 bases 5’ or 3’ to the TSS, respectively [45]. This property allows the ability to form R loop structures during transcription. If transcription is inhibited, the newly transcribed RNA strand anneals to the template DNA strand to form an RNA:DNA hybrid, with the non-template DNA strand existing as ssDNA. ssDNA is subsequently open to the generation of nicks in DNA [46-49] by the action of activation-induced cytidine deaminase (AID) [48, 49], the base excision repair enzymes uracil DNA glycosylase (UNG) and apurinic/apyrimidinic endonuclease (APE), and subsequently DNA DSBs by mismatch repair proteins [49-52].

Both transcription and DNA replication are carried out by the machinery of assembled protein complexes proceeding at DNA templates [53]. Origins of DNA licensing occur in the promoter regions of highly transcribed genes [54, 55], the open chromatin structure favoring the binding of a pre-replication complex (PRC), where origin activity can be stimulated by transcription factors [56]. Thus, replication initiation sites and transcriptionally active sites can be closely linked [54]. Origins of replication are prepared through assembly of PRCs, beginning in late mitosis and continuing through the G1 phase of the cell cycle, with regulated activation of these origins at the G1/S transition [57]. PRC assembly begins when the six-subunit origin recognition complex (ORC1-6) binds to an origin of replication [58]. This is followed by binding of CDC6 to ORC. CDT1, essential for the licensing reaction, binds the core replicative helicase Mini-Chromosome Maintenance complex (MCM) and recruits MCM to DNA replication origins through direct interactions with ORC and CDC6. While both CDC6 and CDT1 are needed to load the MCM complex, they bind in a sequential manner; CDT1 can only bind to chromatin-bound CDC6 and ORC [59]. It has been determined that c-Myc can modulate DNA replication origin activity independent of transcription [60], while c-Myc is also a transcriptional regulator of the licensing factor CDT1 [61].

DNA damage is recognized at the initiation of S-phase [62-64]. Following exposure of HT29 cells to GANT61, a transient intra-S-phase checkpoint is induced and cells accumulate in early S prior to the onset of cell death [22] [65]. FOXM1 is a transcription factor that plays a key role in activating target genes at the G1/S transition [66, 67], is linked to HH signaling in human cancers [68, 69], including colorectal cancer [70], and is an effector of KRAS/BRAF signaling [71]. In this study we demonstrate that FOXM1 is a transcriptional target of GLI1. Following treatment of HT29 cells with GANT61, transcription at the FOXM1 promoter was inhibited by preventing the binding of GLI to chromatin, followed by inhibition of the binding of RNA Pol II and the pause and pause-release factors to the DNA. R-loop formation was decreased by GANT61 with decreased formation of RNA:DNA hybrids and ssDNA in the vicinity of the GLI binding site, suggesting inhibition of GLI-dependent transcription primarily at the PIC. The transcription inhibitor α-amanitin inhibited GANT61-induced DNA DSBs (γH2AX foci), demonstrating the importance of transcription in the induction of DNA damage by GANT61. Through GLI, GANT61 is also involved in the inhibition of DNA replication licensing, which occurs in proximity of the GLI binding site at the FOXM1 promoter. Enrichment of ORC4 binding to chromatin and direct interaction of GLI1 and ORC4 were demonstrated, inhibited by GANT61. Further, we determined that the DNA replication licensing factor, CDT1, present in the DNA licensing complex, is a transcriptional target of GLI1. When overexpressed in HT29 cells, CDT1 reduced caspase-3 cleavage and induction of cell death following treatment with GANT61. Thus, the GLI transcription factor may be the catalyst for both transcription and DNA replication licensing *via* the GLI binding site in promoters of GLI-dependent target genes.

## RESULTS

### FOXM1 is a transcriptional target of GLI

**Figure 1:**
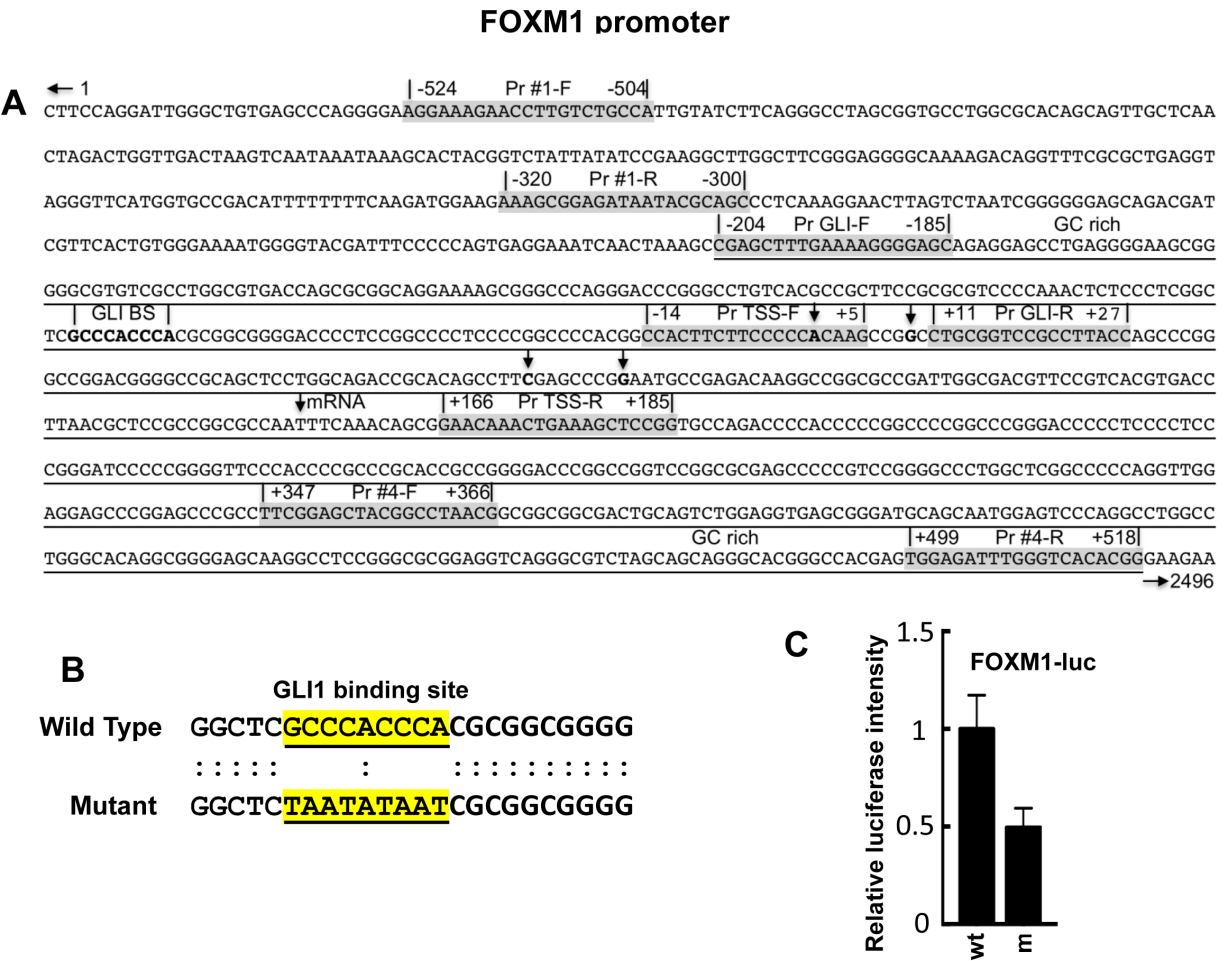
Promoter region of the FOXM1 gene [76] **A.** A putative GLI consensus sequence, GCCCACCCA, was located -54 nucleotides upstream of the first putative TSS. Reported TSS are indicated by vertical arrows. Four primer pairs (Pr #1, Pr GLI, Pr TSS, Pr #4) are shown for both F and R primers to flank four different regions in the FOXM1 promoter; **B.** The putative GLI binding site in a FOXM1 promoter luciferase reporter construct was mutated as described in Materials and Methods; **C.** Co-transfection of the FOXM1 promoter luciferase reporter FOXM1-luc or FOXM1m-luc, pRLTK, and pBabe-Puro into HT29 cells using CalFectin; 24 hr post-transfection, cells were harvested and luciferase activity determined as described in Materials and Methods (*n* = 3).

FOXM1 is known to function downstream of GLI [68], although whether it is directly regulated by GLI has not been determined. The sequence GACCACCCA is the consensus sequence for GLI binding at promoter regions in GLI-dependent target genes [72, 73], with some reported variations [72-75]. In the FOXM1 promoter, a variation of the GLI consensus sequence, GCCCACCCA, is present (Figure 1A). Using a FOXM1 promoter luciferase reporter, the GLI binding site was mutated using the sequence TAATATAAT (Figure 1B). HT29 cells, expressing constitutively activated GLI [23], were transiently co-transfected with Renilla luciferase (pRLTK), pBabe-Puro and FOXM1-luc or mFOXM1-luc for 24 hr. Cells were harvested and assayed for luciferase activity. Following mutation of the GLI binding site, a 50% decrease in FOXM1 promoter luciferase reporter activity was obtained (Figure 1C).

### ChIP analysis demonstrates inhibition of binding of GLI1, Pol II, DSIF, NELF and p-TEFb at the FOXM1 promoter in HT29 cells following GANT61 treatment

**Figure 2:**
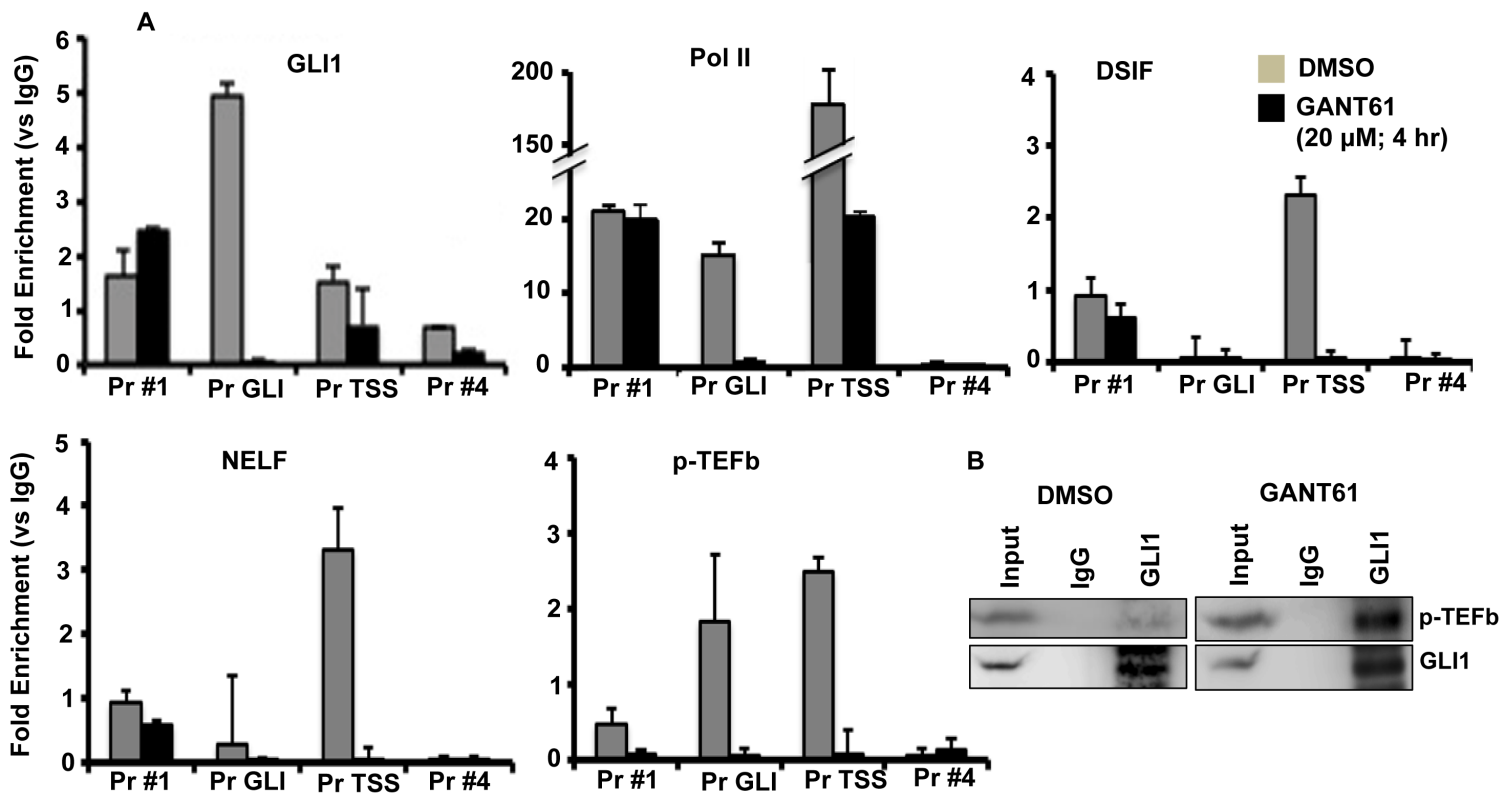
Inhibition of binding of GLI1, Pol II, DSIF, NELF and p-TEFB to the FOXM1 promoter following GANT61 exposure **A.** HT29 cells were treated with GANT61 (20 μM, 4 hr) and chromatin was isolated. ChIP analysis employed antibodies for immunoprecipitation specific for GLI1, Pol II, DSIF, NELF, p-TEFb(cdk9) and IgG (negative control). Subsequent qPCR used primers that flanked four regions of the FOXM1 promoter, including the GLI binding region and the region flanking the TSS. Data (duplicate determinations) are representative of 3 individual experiments. **B.** Co-immunoprecipitation between GLI1 and p-TEFb(cdk9). Experiments were conducted as described in Materials and Methods.

HT29 cells were treated for 4 hr with GANT61 (20 mM); cells were harvested, and chromatin isolated. ChIP analysis employed immunoprecipitation with antibodies (Abs) against GLI1, Pol II, DSIF(Spt5), NELF or P-TEFb(cdk9) followed by qPCR using 4 primer sets designed at the FOXM1 promoter: Pr #1 (-524 to -300, upstream of the GLI binding site), Pr GLI (-204 to +27, flanking the GLI consensus sequence), Pr TSS (-14 to +185, flanking putative TSS, [76]), and Pr #4 (+347 to +518, flanking a region of mRNA; Figure 1A); numbering was according to the first putative TSS. Inhibition of GLI1 binding to the FOXM1 promoter at the GLI consensus sequence by GANT61 was followed by inhibition of binding of the pause factors DSIF and NELF, the release factor p-TEFb in the region of the TSS, and inhibition of binding of Pol II at both sites (Figure 2A). With inhibition of GLI1 binding to chromatin at the GLI consensus sequence being the initiating event in transcriptional inhibition by GANT61, it was anticipated that inhibition of binding of Pol II, DSIF, NELF and p-TEFb would follow. P-TEFb was bound to chromatin in the regions of the GLI consensus sequence and the TSS; binding in both regions was inhibited following treatment of HT29 cells with GANT61 (Figure 2A). In co-immunoprecipitation experiments, p-TEFb and GLI1 demonstrated protein-protein interaction, that was not inhibited by GANT61 (Figure 2B).

### R-loop structures are reduced in HT29 cells following GANT61 treatment

**Figure 3:**
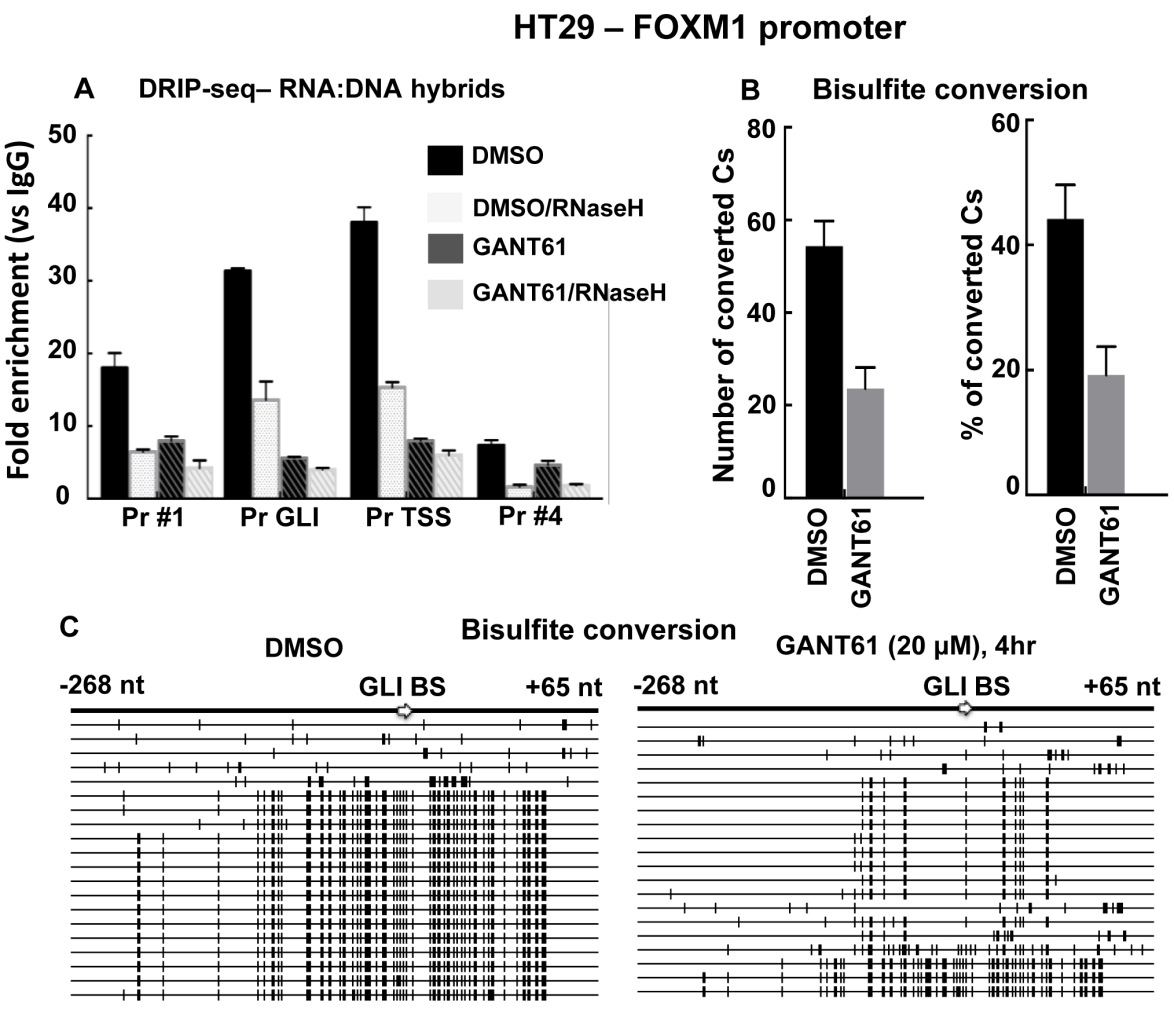
Analysis of R-loop regions at the FOXM1 promoter in the presence or absence of GANT61 **A.** HT29 cells were either untreated (DMSO 0.2% control), or treated with GANT61 (20 μM, 4 hr) and chromatin was isolated. Additional samples were treated with RNaseH to remove RNA:DNA hybrids. Immunoprecipitation was conducted with the S9.6 Ab to pull down RNA:DNA hybrids. The four regions of the FOXM1 promoter were subsequently analyzed by qPCR as described in Materials and Methods. Results are the mean +/- SD of 3 determinations. **B.** Single strandedness of the non-template DNA strand at R-loop regions determined by bisulfite sequencing. HT29 cells were either untreated (DMSO 0.2% control), or treated with GANT61 (20 μM, 4 hr) and genomic DNA was isolated. Bisulfite modification followed by PCR amplification converted C bases to T. PCR products were cloned and sequenced. The region that flanked the GLI binding site was analyzed. Number of converted Cs and % conversion are shown (*n* = 3 +/- SD). **C.** SSDNA. Each long horizontal line represents a single clone; 20 clones for both untreated (DMSO control) or GANT61-treated groups were analyzed. Short bold vertical marks indicate the C residues that converted to U and then to T. The nucleotide positions relative to the GLI binding site are shown.

To determine whether prolonged R-loop formation was involved in the inhibition of GLI-dependent transcription by GANT61, a DNA:RNA immunoprecipitation (DRIP) method [45, 77] was employed using the S9.6 Ab, to specifically immunoprecipitate RNA:DNA hybrids [45, 78]. HT29 cells were untreated, treated with GANT61 (20 mM; 4 hr), or pretreated with RNaseH to degrade the RNA moiety of the RNA:DNA hybrid [79]. Following isolation of chromatin and immunoprecipitation with S9.6, qPCR was conducted using the 4 primer pairs (Figure 1A) to evaluate RNA:DNA hybrid formation at different sites within the FOXM1 promoter (Figure 3A). RNA:DNA hybrids were detected in untreated cells in the regions of the GLI consensus sequence and the TSS, consistent with the binding of Pol II. Following treatment of HT29 cells with GANT61, RNA:DNA hybrid formation was significantly decreased in both regions, consistent with the inhibition of GLI-dependent transcription at the PIC. These structures were also sensitive to pretreatment with RNaseH (Figure 3A).

**Figure 4:**
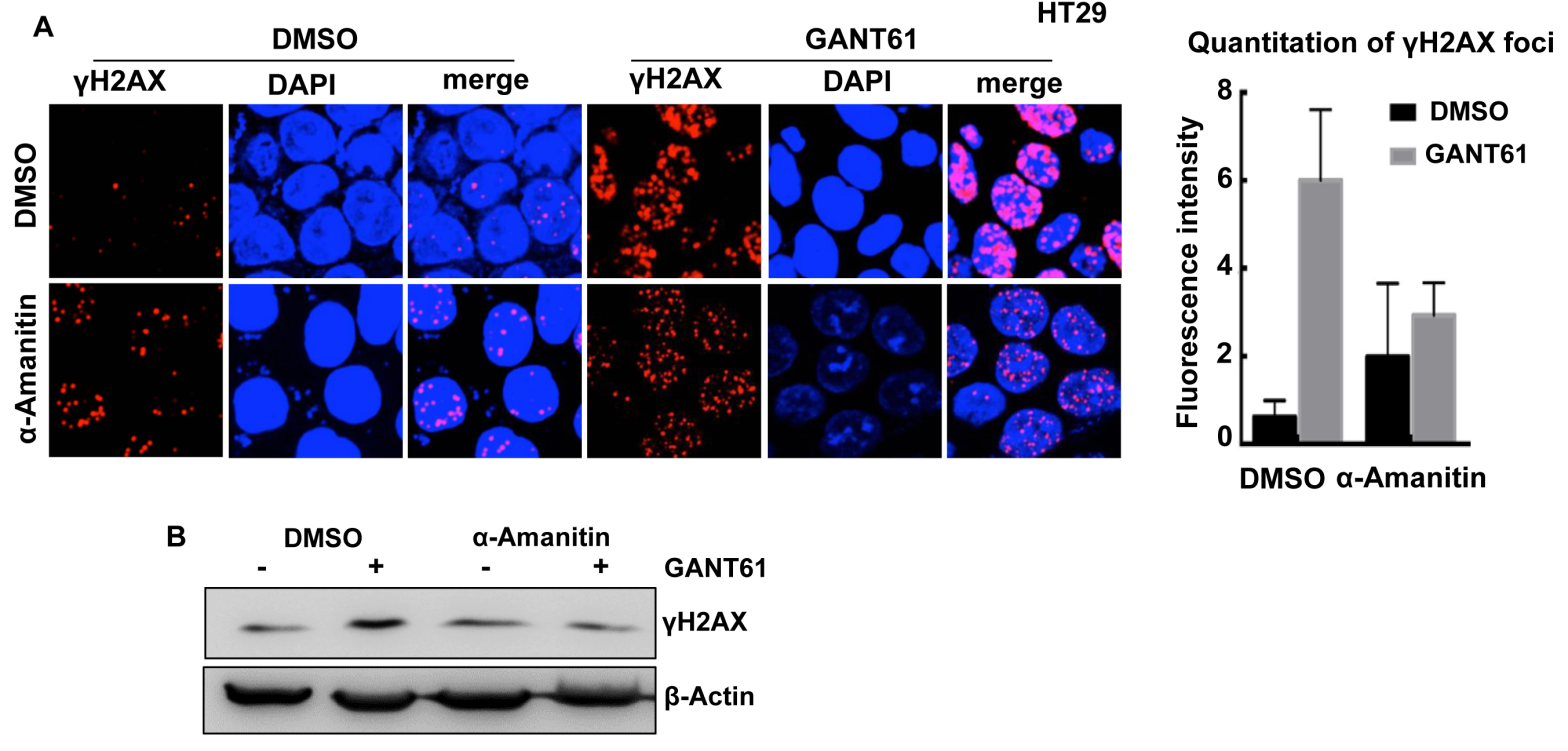
Effect of the Pol II inhibitor α-amanitin on GANT61-induced γH2AX foci HT29 cells were untreated (DMSO 0.2%), or treated with α-amanitin (2 μg/ml) for 2 hr prior to exposure to DMSO (0.2%) or GANT61 (20 μM) for a further 4 hr. **A.** The extent of GANT61-induced γH2AX foci formation was determined by confocal microscopy in the absence of presence of α-amanitin to inhibit transcription; **B.** γH2AX foci in 10-15 cells were quantitated by fluorescence intensity; **C.** γH2AX expression was determined by western analysis.

Since RNA:DNA hybrid formation was reduced in GANT61-treated cells, then treated cells should also demonstrate reduced presence of ssDNA. To confirm this, genomic DNA from control (DMSO 0.2%) or GANT61-treated cells, was isolated. Following bisulfite conversion, the DNA was purified, amplified using conventional PCR and primers that flanked the GLI binding region, which was evaluated in detail. PCR products were purified, cloned, and sequenced. Overall in GANT61-treated cells, bisulfite conversion of C bases was reduced by 50% (Figure 3B). Of the 20 clones sequenced in each of the control and GANT61-treated groups, the majority (75%; 15/20), demonstrated frequent C to T conversion in the control group (Figure 4). In contrast, 85% (17/20) clones demonstrated minimal C to T conversion in GANT61-treated cells in proximity of the GLI binding region, paralleling the reduction in DNA:RNA hybrid formation following GANT61 treatment. These data, together with results from the ChIP analysis, suggest that inhibition of GLI binding by GANT61 at the formation of the PIC determines inhibition of transcription at the FOXM1 promoter, and that prolonged R-loop formation does not play a major role.

To determine the effect of inhibiting GLI-dependent transcription on the induction of DNA damage by GANT61, HT29 cells were pretreated with a specific inhibitor of Pol II-mediated RNA synthesis, α-amanitin (2 μg/ml, [60, 80]) for 2 hr prior to exposure to α-amanitin + GANT61 (20 μM) for 4 hr (Figure 4). While α-amanitin alone caused a slight increase in γH2AX foci, it inhibited the GANT61-induced increase in γH2AX foci (Figure 4A), and γH2AX expression (western analysis; Figure 4B), suggesting a transcriptional component in GANT61-induced DNA damage.

### GLI1 interacts with DNA licensing factors at the PRC in HT29 cells

**Figure 5:**
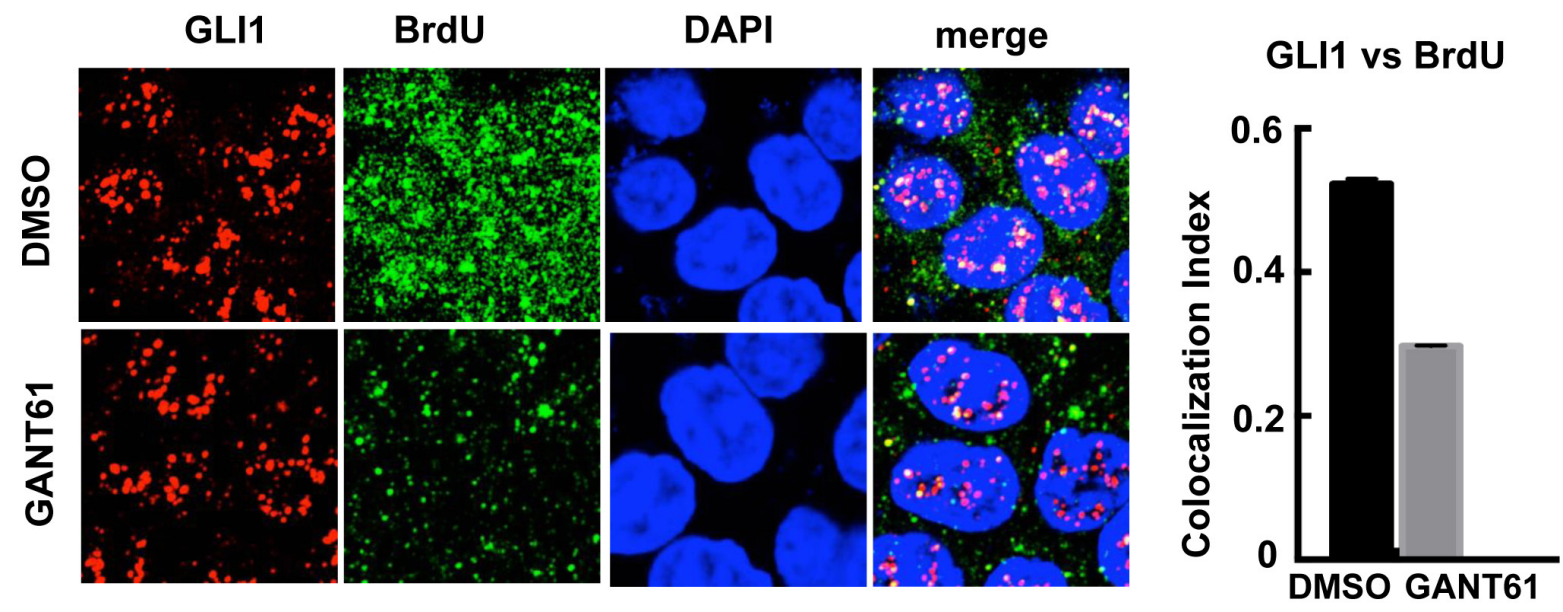
Confocal microscopy of GLI1 expression *vs* incorporation of BrdU into DNA HT29 cells were exposed to DMSO (0.2%) without or with GANT61 (20 μM) for 4 hr, with BrdU (10 μM) added during the final 1 hr of treatment. The co-localization index of GLI1 and BrdU foci was determined using ImageJ (*n* = 10 cells).

Previously we determined by FACS analysis that HT29 cells treated with GANT61 accumulated at the G1/S interface, very early in S-phase [65]. We therefore determined whether GLI1 and BrdU may co-localize at sites of DNA synthesis. This was examined by confocal microscopy both before and after treatment of HT29 cells with GANT61 (20 μM) for 4 hr, with a 1 hr pulse of BrdU (10 μM) prior to harvesting cells. There was significant overlap of GLI1 and BrdU foci, reduced in the presence of GANT61 (Figure 5).

**Figure 6:**
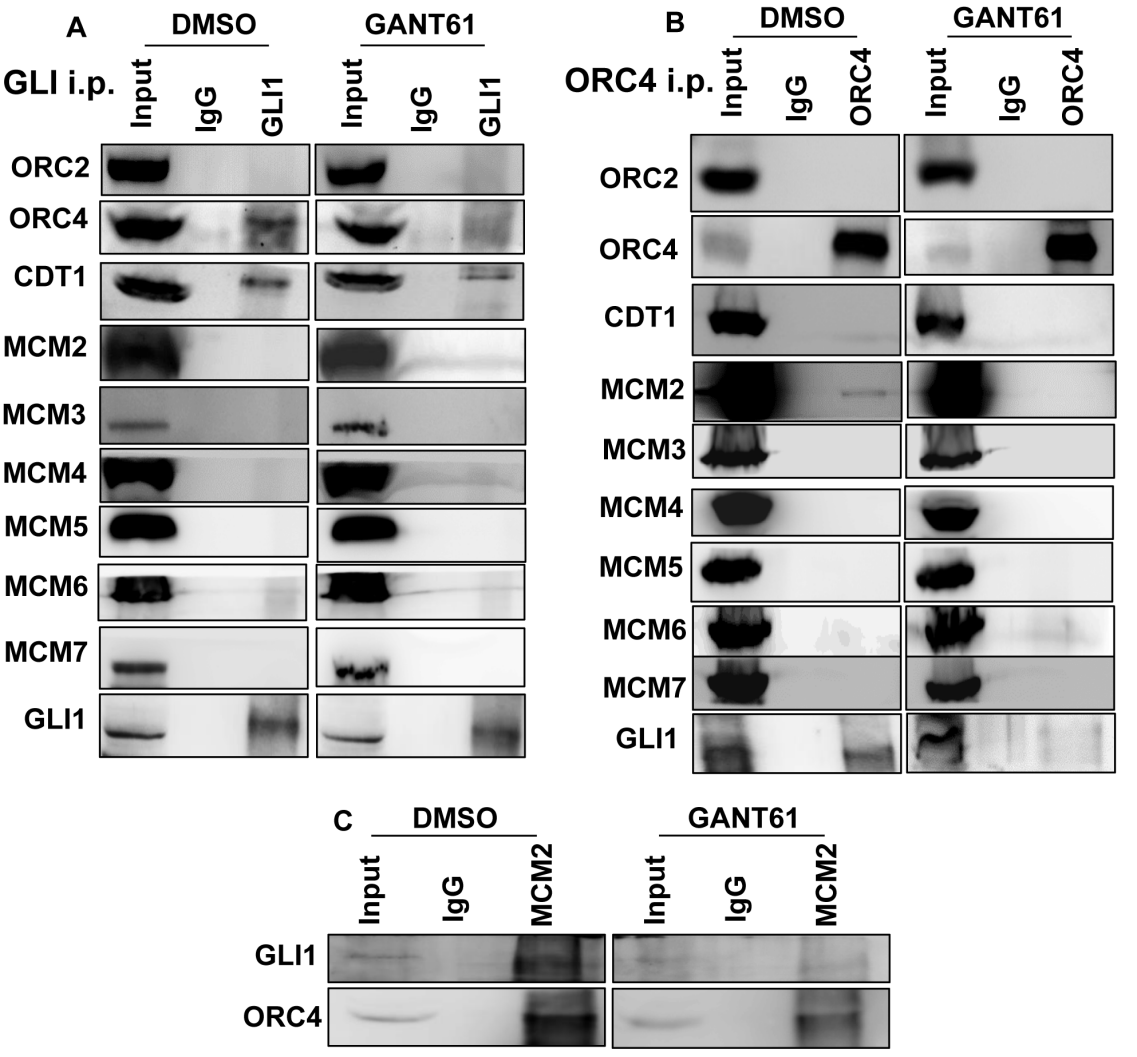
Co-immunoprecipitation of DNA replication licensing factors with GLI1 in GANT61-treated cells Untreated (DMSO 0.2%) or GANT61-treated (20 μM, 4 hr) cells were lysed, cell extracts prepared, and immunoprecipitation conducted with antibodies specific for **A.** GLI1, **B.** ORC4 or **C.** MCM2, as described in Materials and Methods. Western analysis probed for co-immunoprecipitation with GLI1, ORC2, ORC4, CDT1 and MCM2-7.

Transcription and DNA licensing may be coordinately regulated at promoters involving specific transcription factors [56, 81] including c-Myc, which can interact with components of the PRC [60]. The possibility for interaction of GLI with DNA licensing factors was examined. HT29 cells were untreated or treated with GANT61 (20 μM) for 4 hr, cells were harvested, lysed, and cell extracts immunoprecipitated with Abs specific for GLI1 or IgG. Interaction of GLI1 with ORC2, ORC4, CDT1 or MCM2-7, was determined by western analysis (Figure 6). ORC4 and CDT1 co-immunoprecipitated with GLI1. Binding of both proteins to GLI1 was reduced following treatment with GANT61 (Figure 6A). Proteins that co-immunoprecipitated with ORC4 were also determined following immunoprecipitation with an ORC4-specific Ab. GLI1 and MCM2 binding to ORC4 were detected, inhibited by GANT61 (Figure 6B). To confirm the involvement of MCM2 in the GLI1 complex, immunoprecipitation with MCM2-specific Ab detected both GLI1 and ORC4 as binding partners, inhibited by GANT61 (Figure 6C).

### GLI1 binds at an origin of DNA licensing

**Figure 7:**
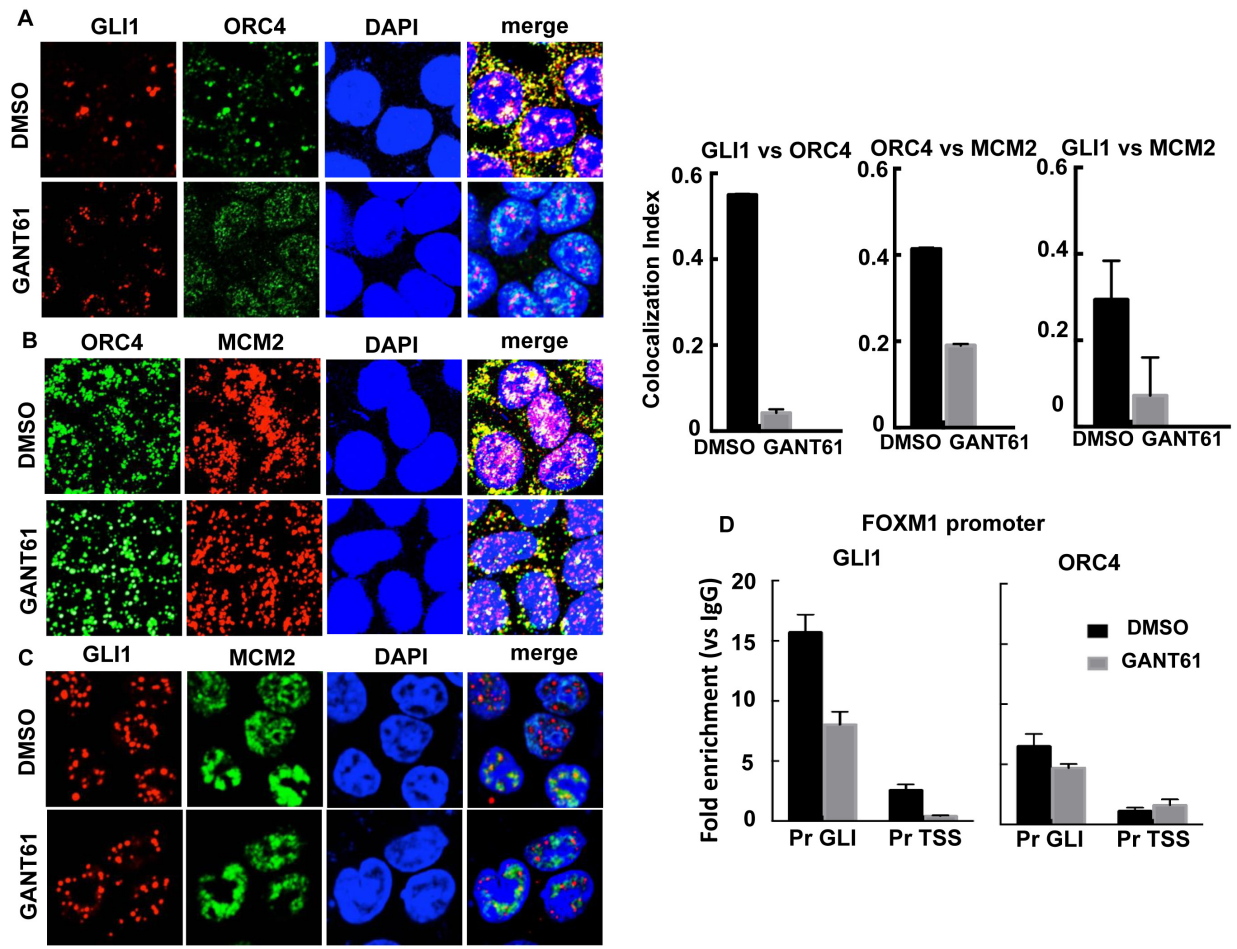
The interaction between GLI1 and DNA licensing factors was examined by confocal microscopy in GANT61-treated cells HT29 cells were exposed to DMSO (0.2%) without or with GANT61 (20 μM) for 4 hr. Foci for GLI1, ORC4 and MCM2 were determined using specific antibodies and the co-localization index determined using ImageJ (*n* = 10 cells). **A.** The co-localization index determined significant interaction between GLI1 and ORC4 in GANT61-treated cells, inhibited by GANT61. GANT61 also decreased the co-localization index for **B.** ORC4/MCM2 and **C.** GLI1/MCM2; **D.** ChIP analysis using antibodies specific for GLI1 or ORC4 followed by qPCR using primers that flanked the GLI binding or TSS regions at the FOXM1 promoter. The analysis determined significant binding of ORC4 in the GLI binding region as well as binding of GLI1, reduced in the presence of GANT61.

Co-localization of GLI1 with ORC4 and MCM2 was examined by confocal microscopy in HT29 cells both before and after treatment with GANT61 (20 μM) for 4 hr (Figure 7). There was significant co-localization of GLI1 and ORC4 foci. This interaction was completely inhibited by treatment of HT29 cells with GANT61 (Figure 7A). ORC4 and MCM2 foci (Figure 7B) as well as GLI1 and MCM2 foci (Figure 7C) also showed a level of co-localization, which was reduced in the presence of GANT61. The binding of ORC4 to the FOXM1 promoter at the proximity of the GLI consensus binding sequence was subsequently evaluated by ChIP analysis and compared to the binding of GLI1 at the same site (Figure 7D). Enrichment of ORC4 on chromatin in the region of the GLI binding sequence was determined. Both GLI1 and ORC4 demonstrated inhibition of binding to chromatin following treatment of HT29 cells with GANT61. Data demonstrate a significant interaction of GLI1 with DNA licensing factors by a non-transcriptional mechanism, in particular between GLI1 and ORC4.

### CDT1 is a transcriptional target of GLI1

**Figure 8:**
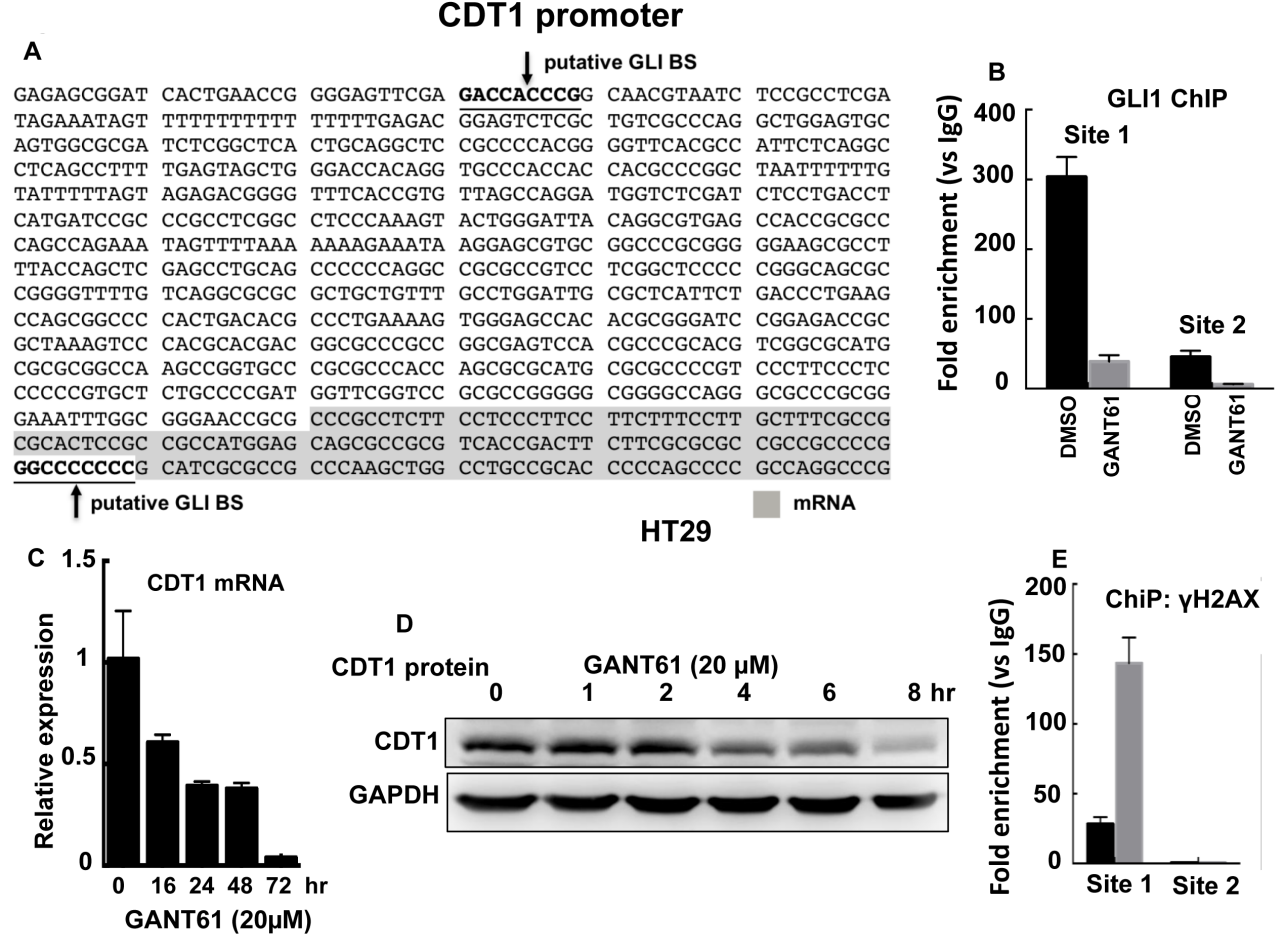
CDT1 is a transcriptional target of GLI1 **A.** The CDT1 promoter with two putative GLI binding sites; **B.** ChIP analysis of GLI1 binding at the CDT1 promoter in HT29 cells demonstrates GLI1 binding at Site 1 and not Site 2; CDT1 mRNA expression **C.** and protein **D.** rapidly decrease in the presence of GANT61 (20 μM); **E.** ChIP analysis demonstrates enrichment of γH2AX foci at Site 1 of the CDT1 promoter in HT29 cells following GANT61 treatment (20 μM, 4 hr).

We determined that in contrast to ORC4 and MCM2, CDT1 harbored two putative GLI binding sequences in the CDT1 promoter: GACCACCCG (site 1, promoter) and GGCCCCCCC (site 2, mRNA), suggesting that GLI may transcriptionally regulate CDT1 (Figure 8A). ChIP analysis employed GLI1 immunoprecipitation and DNA sequencing using primers that flanked each of the two putative GLI binding sequences. GLI1 bound to the putative GLI binding sequence on chromatin at site 1 but not at site 2. GLI1 binding at site 1 was significantly inhibited in GANT61-treated HT29 cells (Figure 8B). CDT1 mRNA was rapidly decreased in GANT61-treated HT29 cells, reduced to almost 50% by 16 hr, and undetectable by 72 hr (Figure 7C). Further, CDT1 protein rapidly decreased in GANT61-treated HT29 cells, with low-level expression by 8 hr (Figure 8D). Following ChIP analysis by immunoprecipitation of γH2AX at the CDT1 promoter, significant enrichment of γH2AX at the functional GLI binding site, site 1, was demonstrated (Figure 8E).

### Role of DNA replication licensing in GANT61-induced cell death

**Figure 9:**
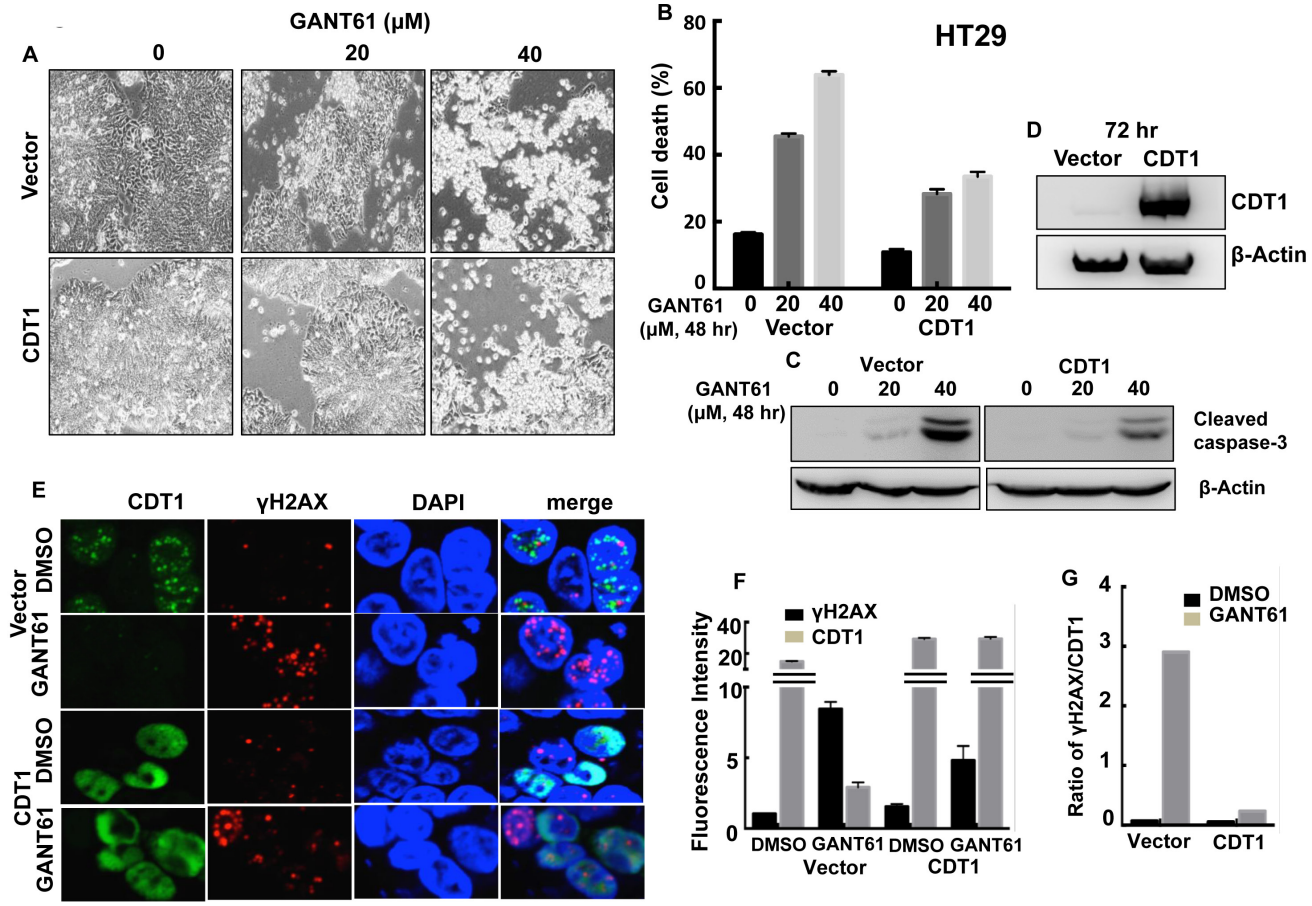
CDT1 overexpression in HT29 cells during exposure to GANT61 Transient transfection of HT29 cells with CDT1 for 24 hr prior to treatment with GANT61 (20 μM, 40 μM) for 48 hr. **A.** Cells were examined under an inverted microscope at 72 hr after transient transfection to determine cellular morphology; **B.** induction of cell death, or **C.** cleavage of caspase-3 following GANT61 treatment for 48 hr; **D.** expression of CDT1 determined by western analysis following transient transfection for 72 hr in HT29 cells; **E.** GANT61-induced γH2AX foci were determined by confocal microscopy in the absence or presence of CDT1 overexpression; **F.** Fluorescence intensity of γH2AX foci in cells overexpressing CDT1 compared to vector control in the absence or presence of GANT61; **G.** Ratios of γH2AX/CDT1 in cells +/- CDT1 overexpression and +/- GANT61.

**Figure 10:**
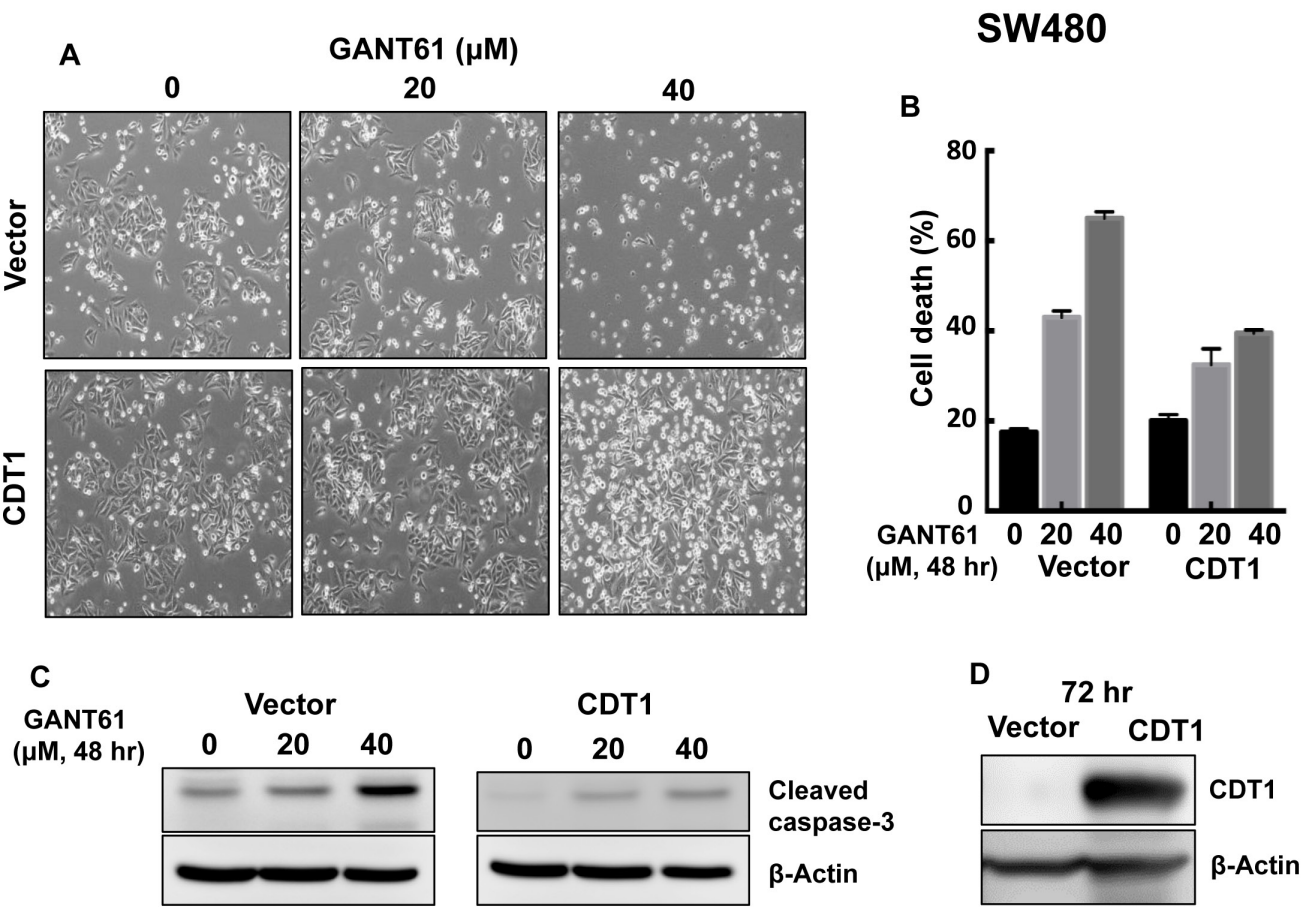
CDT1 overexpression in SW480 cells and treatment with GANT61 were conducted as described in the legend to Figure 9 **A.** cellular morphology; **B.** induction of cell death; **C.** cleavage of caspase-3; **D.** expression of CDT1.

To elucidate the role of DNA licensing in GLI-dependent, GANT61-induced cell death, CDT1 was transiently transfected for 24 hr prior to treatment of HT29 cells with GANT61 (20 μM, 40 μM) for 48 hr (Figure 9A). The extent of cell death was determined by FACS analysis following Annexin V-FITC/PI staining (Figure 9B). In the presence of CDT1 overexpression, GANT61-induced cell death was significantly reduced. Similarly, GANT61-induced cleavage of caspase-3 was reduced (Figure 9C) when CDT1 was overexpressed (Figure 9D). The induction of γH2AX foci by GANT61 was examined by confocal microscopy in the absence or presence of CDT1 overexpression (Figure 9E), which significantly reduced GANT61-induced γH2AX foci (Figure 9F), as well as the ratio of γH2AX /CDT1 (Figure 9G). Similar results were obtained in SW480 human colon carcinoma cells (Figure 10). Data indicated that CDT1 expression, DNA damage, and GANT61-induced cell death are intricately linked.

## DISCUSSION

We have previously demonstrated rapid inhibition of binding of the GLI1 and GLI2 transcription factors to target gene promoters (1 hr), reduced reporter activity specific to GLI-luciferase, and rapid inhibition of gene transcription in human colon carcinoma cell lines in response to GANT61 [65]. GANT61 induces extensive cytotoxicity in human colon carcinoma cell line models following inhibition of GLI-dependent transcription [21-23]. Overexpression of GLI1 or GLI2 also protects cells from GANT61-mediated cell death [21]. Binding of transcription factors to DNA takes place during all phases of the cell cycle, except during mitosis [25, 26]. GANT61 induces γH2AX foci by an ATM-CHK2-dependent signaling cascade with induction of a transient intra-S-phase checkpoint when cycling cells reach G1/S. Progression beyond early DNA synthesis is inhibited, GLI-dependent target genes that regulate the G1/S transition and DNA replication are downregulated, and cell death ensues [22, 23, 65, 82]. GLI is a nodal point of activation for oncogenic signaling pathways, including oncogenic KRAS/BRAF, which is channeled through GLI, driving GLI to a higher activating state [22]. The binding of GANT61 is specific for GLI, with no detected binding to DNA or to other transcription factors [24]. Thus, GLI is a critical gene that determines survival in tumors with constitutive GLI activation and/or oncogenic KRAS signaling, and suggests that inhibition of GLI-dependent transcription plays a major role in the induction of cell death.

Pol II executes a series of distinct steps during transcription by binding to promoters that requires specific as well as general transcription factors. RNA synthesis is initiated, and pausing during early transcriptional elongation follows. Pol II accumulates disproportionately at promoters [31, 83, 84], concentrated near the TSS [29, 84, 85]. Paused Pol II remains stably associated with the nascent RNA until further signals facilitate transition to an elongation complex. Initially it was thought that minimal transcriptional regulation occurred after formation of the PIC [86], however more recent findings suggest additional regulation of the transcription machinery can occur at the site of pause-release [39, 86]. Key cell regulatory genes including c-Myc and Fos have demonstrated enrichment of bound Pol II just downstream of the TSS [87, 88]. DSIF and NELF are both required for promoter proximal pausing, subsequently eliminated by p-TEFb [39]. It has been suggested that the rate of pause-release may be regulatory for transcription [33]. In the current study, GANT61 inhibited binding of GLI1 to DNA in the region of the GLI consensus sequence at the FOXM1 promoter. Following inhibition of GLI1 binding, Pol II binding to chromatin was inhibited in the areas of the GLI binding site and the TSS. Binding of DSIF, NELF and p-TEFb were all inhibited downstream of the GLI binding sequence, in the region of the TSS, consistent with their regulatory functions in transcription pausing or pause-release. The presence of p-TEFb in the region of GLI binding is consistent with its ability to co-immunoprecipitate with GLI1. It has been suggested that binding of c-Myc [30] or NF-κB [41] to p-TEFB stimulates transcriptional elongation by Pol II at the site of pause-release. However GLI1/p-TEFB binding was not inhibited in the presence of GANT61. Further, if p-TEFb was the target of inhibition of GLI-dependent transcription by GANT61, it would be anticipated that Pol II would remain bound at the PIC at the GLI binding domain, but be inhibited at the DSIF/NELF/p-TEFb promoter-proximal pause site; however, this was not the case. Thus following GANT61 treatment, inhibition of GLI binding to chromatin at the PIC also inhibited Pol II binding at the GLI binding region.

Promoter regions in actively transcribed genes harbor GC rich sequences that promote the formation of R-loops during transcription [45]. Long R-loop structures frozen during inhibition of GLI-dependent transcription by GANT61 could facilitate the introduction of strand breaks in DNA at fragile C bases by enzymatic conversion if transcription is inhibited [48-52]. In the current study RNA:DNA hybrids were found to be reduced in GANT61-treated cells in particular in the regions of the GLI binding site and TSS, confirmed by their sensitivity to RNaseH. These data were supported by reduction in the presence of ssRNA in the region of the GLI binding site that was studied in detail. Findings suggest GANT61-induced inhibition of GLI-dependent transcription at a point early in transcription, during initiation of formation of the PIC, that does not allow elongation to proceed, and hence reduces R-loop formation. The importance of transcriptional inhibition in the mechanism of GANT61 action was demonstrated from the findings that α-amanitin, an inhibitor of Pol II, significantly inhibited GANT61-induced induction of DNA damage determined by reduction in GANT61-induced γH2AX foci and expression.

CpG islands in promoter regions are initiation sites for both transcription and DNA replication [44]. In mammalian cells, transcription and replication complexes can meet at specific genomic positions [54]. Because replication origins are usually enriched within promoter regions within CpG sequences, the open chromatin structure at such sites can favor the binding of a replication pre-initiation complex [54]. Transcription factors at sites of replication initiation have been shown to stimulate replication in many systems [56, 81]. Similar to RNA polymerases, DNA polymerases have no intrinsic ability to recognize specific DNA sequences on promoters [53]. Therefore DNA replication initiation and RNA transcription initiation share mechanisms that recruit polymerases by an orderly assembled protein complex [53]. An important question is how transcription and replication are coordinated at the chromatin level to allow flexibility for transcription and replication to proceed in a coordinated manner [89]. We demonstrated previously by FACS analysis in HT29 cells that GANT61-treated cells accumulated at the G1/S boundary, with minimal incorporation of BrdU [65]. In the current study we determined that significant co-localization of GLI1 and BrdU foci were reduced in the presence of GANT61, suggesting that GLI1 might be present at or near replication origins. We have now demonstrated that there are protein-protein interactions among GLI1, ORC4, MCM2 and CDT1, and significant co-localization of GLI1 and ORC4 foci, inhibited by GANT61, suggesting their interaction at an origin of DNA replication licensing. We demonstrated by ChIP analysis enrichment of ORC4 at the GLI consensus site in the FOXM1 promoter, also suggesting that GLI is localized to early sites of DNA synthesis. It has been reported that c-Myc can interact with MCM2 and MCM7 [90]. Further, all MCM2-MCM7 subunits, ORC2, CDC6 and CDT1 were present in an affinity-purified Myc complex, and ORC2 was found enriched at Myc origins on chromatin [60]. In similarity to c-Myc, current findings suggest coordinate regulation of GLI-dependent transcription and DNA replication licensing at foci of GLI binding in promoters.

During examination of the promoters of DNA licensing factors involved in a PRC with GLI1, we identified two putative GLI binding sequences in the CDT1 promoter, not present in the promoters of other molecules. Out of the two binding sites, site 1 only was found to be functional for GLI1 binding. CDT1 mRNA and protein were rapidly decreased following treatment of HT29 cells with GANT61, consistent with inhibition of transcriptional regulation. Further by ChIP analysis, γH2AX was found enriched on chromatin at the functional GLI binding site. The CDT1 promoter has been identified as transcriptionally regulated by c-Myc [61], suggesting that CDT1 may be a key transcriptional target of oncogenes that drive cellular proliferation. By overexpressing CDT1 by transient transfection in HT29 or SW480 cells, exogenous CDT1 reduced the cleavage of caspase-3, significantly rescued GANT61-induced cytotoxicity and DNA damage (γH2AX foci), demonstrating its importance in regulating GANT61-induced cell death.

In this study, we have demonstrated the inhibition of GLI-dependent transcription by GANT61 at the PIC during the initiation process of RNA synthesis, and the coordination of transcription and DNA replication licensing at sites in promoter regions that bind the specific transcription factor GLI1. The involvement of GLI1 in DNA licensing involves: 1) non-transcriptional mechanisms by direct interaction of GLI1 with ORC4 in a complex at the origins (which are also sites of transcription initiation involving GLI), and 2) transcriptional mechanisms that involve transcriptional regulation of the licensing factor CDT1. DNA licensing factors have been identified by others as components of the transcription machinery. MCM proteins have been found in several studies to be involved in RNA transcription [53], implicating the coordination of transcription and replication. MCM2-7 proteins can co-localize with Pol II on actively transcribing genes [91], and move along with Pol II during transcription elongation [92]. MCM2 and MCM5 have also been found to be required for general transcription [53]. Thus, interaction between the transcription and DNA replication licensing machineries are important in regulating mechanisms that control cell survival and cell death in cancer cells.

## MATERIALS AND METHODS

### Cell culture and reagents

The human colon carcinoma cell lines HT29 and SW480 have been described previously [21-23, 65]. Cells were cultured in 10% FBS-supplemented RPMI medium with L-glutamine and maintained at 37°C with 5% CO_2_. Antibodies were obtained from the following sources: GLI1 (ChIP analysis [Novus]; Western analysis [Cell Signaling]), RNA Pol II, DSIF(SPT5), NELF (Santa Cruz), CDT1, p-TEFb (cdk9; Cell Signaling), anti DNA:RNA hybrid S9.6 (Kerafast Inc.), γH2AX (ChIP [Millipore]; IHC [Cell Signaling]), ORC2 (Enzo), ORC4, MCM5 (Abcam), MCM6 (Novus), MCM2, MCM3, MCM4, MCM7 (Cell Signaling); BrdU (BD Pharmingen), cleaved caspase-3 (Cell Signaling).

### Site-directed mutagenesis and FOXM1-luciferase assay

Two complimentary oligonucleotides for mutation of the GLI BS to convert GCCCACCCA to TAATATAAT, were purchased from IDT (Integrated DNA Technologies). The mutation reaction was set up using the QuikChange II Site-Directed Mutagenesis kit (Agilent Technologies Inc.). The reaction mixture contained 50 ng of template DNA (pBabe-GLI1), 125 ng each of the oligonucleotide primer, 200 μM dNTP mix, 2.5 U of PfuUltra HF DNA polymerase and I x reaction buffer in a total volume of 50 μl. The samples were denatured at 95°C for 30 sec and then cycled 16 times at 95°C for 30 sec, 55°C for 1 min followed by 68°C for 9 min. The parental plasmid DNA was digested by adding 10 U of Dpn I restriction enzyme to the amplification reaction and incubated at 37°C for 1 hr. DH5α competent cells (Agilent Technologies) were transformed with 1 μl Dpn I-treated DNA and plated on LB-agar plate containing 100 μg/mL ampicillin. Plates were incubated at 37°C for 16 hr. Single colonies were isolated and plasmid DNA extracted and sequenced for mutation verification.

The FOXM1 promoter luciferase reporter (FOXM1-luc) containing the whole sequence of the FOXM1 promoter was a gift from Dr. Suyun Huang, MD Anderson Cancer Center. FOXM1-luc (2 μg) or FOXM1m-luc (2 μg) and Renilla luciferase (0.2 μg, pRLTK) were cotransfected with pBabe-Puro (2 μg, empty vector) into HT29 cells for 24 hr using CalFectin Mammalian DNA Transfection Reagent (SignaGen Laboratories). Cells were subsequently harvested using the Dual luciferase reporter assay system (Promega Corporation) according to the manufacturer's protocol. Luciferase activity was detected by a Victor2 multilabel counter and normalized to Renilla luciferase activity as a control for transfection efficiency.

### ChIP analysis

HT29 cells (2x10^6^) were seeded in T75 flasks. After overnight attachment, cells were treated with DMSO (0.2%) or GANT61 (20 μM) for 4 hr. The cells were trypsinized, washed with 1x PBS and fixed with 1.1% formaldehyde for 10 min at RT. Glycine (0.125M) was added to stop the reaction. Cells were washed with PBS x 1 and lysed using the ChIP kit according to the manufacturer's instructions (Abcam, Ab500). To fragment the DNA, the cells were sonicated (30 sec with 30 sec cooling repeatedly for 15 cycles) to obtain fragmented DNA from 100 bp to 1 kB which was verified by agarose gel migration. Lysates were subjected to immunoprecipitation overnight at 4°C with 10 μg of anti-human antibodies for GLI1, RNA Pol II, DSIF, NELF, p-TEFb, ORC4, CDT1, MCM2, γH2AX and Histone H3 for positive control provided by the Abcam ChIP kit. The complex was subsequently incubated with Dynabeads protein G (Invitrogen) for 2 hr, 4°C, on a rotating wheel. Beads were washed, 10 min, 4°C with low salt buffer (2x TE, 150mM NaCl, 1% Triton X-100, 0.1% SDS); 10 min. 4°C, with high salt buffer (2x TE, 500mM NaCl, 1% Triton X-100, 0.1% SDS); 10 min, 4° with LiCl buffer (1x TE, 1% NP-40, 0.25M LiCl, 1% deoxycholate); and finally for 5 min, 4°C x 2 with TE Buffer (100mM Tris- HCl, 10mM EDTA) and finally eluted by heating for 10 min at 70°C with elution buffer, and incubation at 65°C overnight. Purification of DNA was accomplished using the QIAquick PCR purification kit (QIAGEN) according to the manufacturer's directions. Quantitative realtime PCR (qPCR) was performed to access the enrichment of the specific proteins along the FOXM1 promoter using specific primer pairs: Primer #1-F: AGGAAAGAACCTTGTCTGCCA (-524 to -504), Primer #1-R: GCTGCGTATTATCTCCGCTTT (-320 to -300); Pr GLI-F: CGAGCTTTGAAAAGGGGAGC (-204 to -185), Pr GLI-R: GGTAAGGCGGACCGCAG (+11 to +27); Pr TSS-F: CCACTTCTTCCCCCACAAG (-14 to +5); Pr TSS-R: GAACAAACTGAAAGCTCCGG (+166 to +185); Pr #4-F: TTCGGAGCTACGGCCTAACG (+347 to +366), Pr #4-R: CCGTGTGACCCAAATCTCCA (+499 to +518), calculated from the first predicted TSS [76]. Two primer pairs were also employed to flank each of the putative GLI binding sites on the CDT1 promoter: CDT1-promotor-1-F: GAGAGCGGATCACTGAACCG, CDT1-promotor-1-R: GAGATCGCGCCACTGCACT; CDT1-promotor-2-F: TCACCGACTTCTTCTTCGCGCGC, CDT1-promotor-2-R: CTTGCGGCTACCACTGGTAG.

### DRIP-Seq

For mapping of RNA:DNA hybrids (R-loops) by DNA-RNA immunoprecipitation (DRIP) and sequencing (DRIP-seq) within the FOXM1 promoter, the procedure utilizes a sequence-independent but structure specific antibody, S9.6; the methodology is essentially as described [45, 77]. HT29 cells were plated as for ChIP analysis, and after overnight attachment were treated with DMSO (0.2%) or GANT61 (20 μM) for 4 hr. Cells were harvested as above, and genomic DNA extracted using the mini prep kit (Qiagen) prior to storage at -20°C. S9.6 Ab (10 μg) was mixed with 50 μl protein G beads (Life Technologies) at 4°C overnight. DNA was fragmented by sonication as described above. For immunoprecipitation, samples (100 μl) were processed, in triplicate, for input control, DMSO control, DMSO treated with 10 μl RNaseH (NEB Inc.; overnight), or IgG negative control (10 μg mouse IgG, Santa Cruz). RNaseH was employed to degrade the RNA moiety of the RNA:DNA hybrid and hence the R-loop structure as a control [93, 94]. GANT61-treated samples were similarly processed. All samples except input controls were added to the preincubated S9.6 Ab/protein G beads (1 ml in PBS) overnight on a rotator at 4°C. Beads were washed by resuspending in low salt buffer (2x TE, 150 mM NaCl, 1% Triton-X 100, 0.1% SDS, 700 μl), followed by high salt buffer (2xTE, 500 mM NaCl, 1% Triton X-100, 0,1% SDS, 700 μl), and LiCl buffer (1xTE, 1% NP-40, 0.25M LiCl, 1% deoxycholate, 700 μl), each with incubation for 10 min on a rotator at 4°C. Subsequently beads were resuspended twice in TE buffer (700 μl) each for 5 min on a rotator at 4°C. Elution of the RNA:DNA hybrids bound to chromatin was effected using the Ipure kit (Diagenode) according to the manufacturer's protocol. Samples were analyzed directly by qPCR using the FOXM1 promoter primer pairs described for ChIP analysis.

### Bisulfite modification

The assay is essentially as described [45, 47]. Following bisulfite conversion, unpaired C nucleotides on ssDNA are converted to U bases. During subsequent PCR, U bases are converted to T. Genomic DNA from control (DMSO, 0.2%) or GANT61 (20 μM; 4 hr)-treated cells was extracted as above. Bisulfite conversion under non-denaturing conditions was accomplished using the EpiTect Bisulfite kit according to the manufacturer's instructions, except for the bisulfite incubation which was at 37°C overnight. The bisulfite converted DNA was purified according to the kit instructions. Molecular amplification of the deaminated DNA as well as conventional PCR was effected using the HotStar Taq kit (Qiagen) according to the manufacturer's directions. Amplification products were purified on an agarose gel (1%). The PCR products were purified using the Qiagen purification kit and subsequently cloned into the TA cloning vector (Thermo Fisher) according to the manufacturer's instructions. Cloned plasmids were transformed into TOP10 competent cells with blue white screen. Confirmation of the PCR product was verified by PCR, colonies were expanded, the plasmids purified and subsequently sequenced.

### Co-immunoprecipitation

HT29 cells were plated in 150mm dishes at 50% confluency the day before treatment. Cells were treated with DMSO (0.2%) or GANT61 (20 μM) for 4 hr. Media was removed and cells were washed twice with PBS. 200μl of Pierce Lysis buffer (ThermoFisher) supplemented with protease inhibitor cocktail and 1% NP-40 were added to the dish for 30 min on ice. A cold scraper was used to harvest the cells and the lysate was transferred to a cold 1.5 ml microcentrifuge tube. Samples were centrifuged at 13,000 rpm, for 10 min at 4°C to pellet cell debris. The supernatant was transferred to a new 1.5 ml microcentrifuge tube and stored at -20°C before use. For each ip, lysate (50 μl) was transferred to a 1.5 ml microcentrifuge tube. Antibody, or IgG control, was added to each sample and the solution volumed up to 500 μl using TBS supplemented with protease inhibitor cocktail. The samples were incubated overnight at 4°C with rotation. The following day, Dynabead Protein G magnetic beads 60 μl (per ip sample) were washed x 3 with cold TBST. Beads were resuspended with the lysate-antibody solution and incubated for 2 hr at 4°C with rotation, followed by washing x 3 with cold TBST supplemented with protease inhibitor cocktail. Supernatant was removed and the beads resuspended in 2x Laemmli buffer (100 μl) with β-mercaptoethanol. Samples were heat-treated for 10 min at 70°C and placed on ice. Each sample (25 μl), including the Input lysate, was loaded onto a SDS-PAGE gel and processed for Western blot analysis. Co-immunoprecipitated proteins were evaluated including GLI1, p-TEFb, ORC2, ORC4, CDT1 and MCM2-7.

### Confocal microscopy

Cells were plated at a density of 50,000 cells/well in 6-well plates on coverslips and allowed to attach overnight. Media was removed and cells were treated with DMSO (0.2%) or GANT61 (20 μM) for 4 hr. Cells were also pretreated with the RNA Pol II inhibitor α-amanitin (2 μg/ml) for 2 hr prior to treatment with GANT61. Coverslips were removed and placed in a humidity chamber for fixation by absolute methanol for 10 min at 4°C. Permeabilization by acetone was effected for 1 min at 4°C followed by staining. After washing with PBS x 3, cells were incubated with the diluted primary antibody overnight at 4°C. Cells were subsequently washed x 3 and incubated with appropriate secondary antibody at RT in the dark for 1 hr. After washing with PBS, cell nuclei were stained with DAPI at RT for 5-10 min. Confocal images were acquired on a Nikon A1 laser confocal system with a Nikon Eclipse Ti microscope and a 60X Plan Apo objective. Lasers used were 405 nm for blue, 488 nm for green, 561 nm for red. NIS Elements AR 4.5000 software was used to acquire Z-stacks of each channel sequentially to avoid spectral cross talk. Each slice was captured at a 0.15-μm step. BrdU incorporation was determined by incubating cells with BrdU (10 μM) for 1 hr prior to harvesting of cells. Primary antibodies used were GLI1 (Novus 1:500), ORC4 (Abcam 1:500), γH2AX (Millipore 1:500), BrdU (BD Pharmingen 1:500), CDT1 (Cell Signaling 1:500), MCM2 (Cell Signaling 1:500). The co-localization index for specific foci was determined using ImageJ.

### Analysis of cell death

HT29 and SW480 cells were treated, in duplicate, as described in the Figure legends. At the end of treatment, cells were collected by trypsinization and incubated with Annexin V FITC (BD Biosciences) and propidium iodide (Sigma) prior to analysis using a FACSCalibur flow cytometer, as described [21, 23]. Data were analyzed using FlowJo software.
